# Supplementary material for: Cell-selective proteomics segregates pancreatic cancer subtypes by extracellular proteins in tumors and circulation
Source: Nat Commun. 2023 May 8;14:2642. doi: 10.1038/s41467-023-38171-8 (PMC10167354; doi:10.1038/s41467-023-38171-8)
Supplement: Supplementary file 1 — Supplementary Information [file 41467_2023_38171_MOESM1_ESM.pdf]

Supplementary Figures 1-9:

Supplementary Fig. 1: Comparison of Met-substitute incorporation rates in vitro.

Supplementary Fig. 2: Technical reproducibility of MetRS*-based cell-selective proteomics experiments, related to Fig. 1.

Supplementary Fig. 3: Specifically enriched MetRS* 8661 PDAC cell-derived proteins with different enrichment methods, related to Fig. 1.

Supplementary Fig. 4: Technical reproducibility of enrichment specificity controls.

Supplementary Fig 5: Flow cytometry analysis of MetRS*-eGFP 8661 cells, related to Fig. 2.

Supplementary Fig 6: Principal component analysis (PCA) of cell-selective proteomics samples from BMMs and PDAC cells in co-culture and isolation in vitro, related to Fig. 3.

Supplementary Fig 7: Heatmaps of cell-selectively secreted proteins in co-culture experiments, related to Fig. 3

Supplementary Fig. 8: Immunophenotyping of PDAC tumors by flow cytometry, related to Fig. 4.

Supplementary Fig. 9: PCA of PDAC tumor-bearing mouse serum samples, related to Fig. 6.

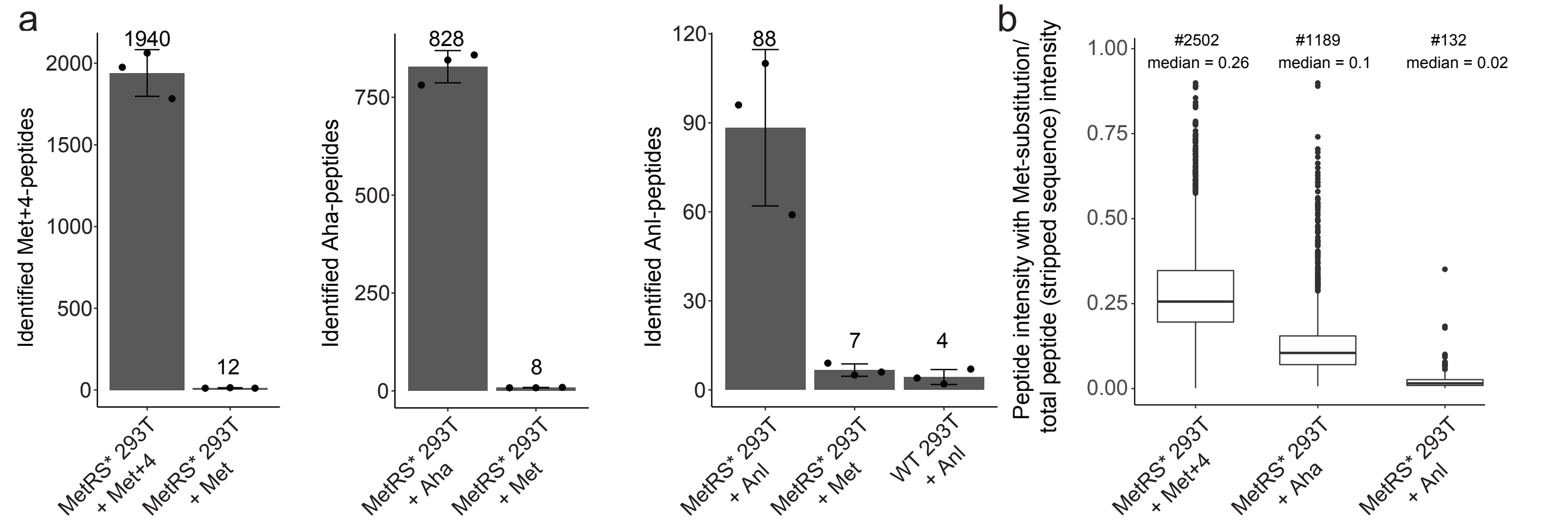

Supplementary Fig. 1: Comparison of Met-substitute incorporation rates *in vitro*.
MetRS*-expressing 293T cells were grown for 8 h in Met-free medium supplemented with 4 mM L-methionine-methyl-^{13}C,d_3 (Met+4), Aha or Anl, and processed for shotgun proteomics and DDA single shot analysis without selective enrichment. **a)** Average number of identified peptides with methionine-substitutions (n = 3 , mean ± SD). Cells grown in 4 mM non-isotopically labeled methionine were used to evaluate false discovery rates, and WT 293T cells incubated in Anl-supplemented media to evaluate unspecific incorporation without MetRS*. **b)** Median intensity ratios of peptides with Met-substitution and all modified and non-modified peptides with the corresponding sequence.

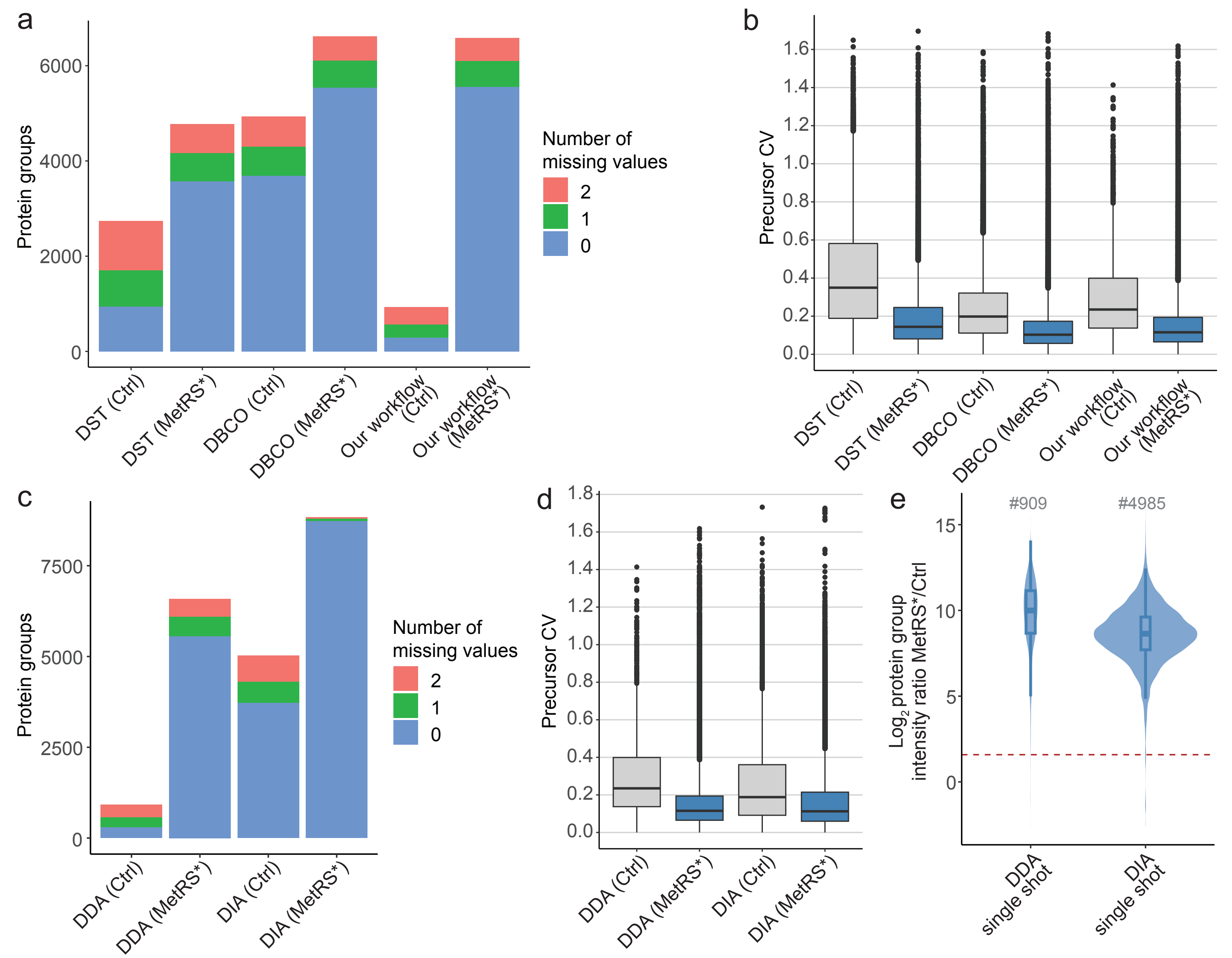

Supplementary Fig. 2: Technical reproducibility of MetRS*-based cell-selective proteomics experiments, related to Fig. 1.
a) Data completeness and **b)** precursor coefficients of variation (CVs) of MetRS* and Ctrl samples processed with different Anl enrichment workflows (see Fig. 1a). **c)** Data completeness, **d)** precursor CVs, and **e)** intensity ratios of proteins identified in both MetRS* and Ctrl samples after processing with our enrichment workflow and MS analysis by data-dependent acquisition (DDA) or data-independent acquisition (DIA).

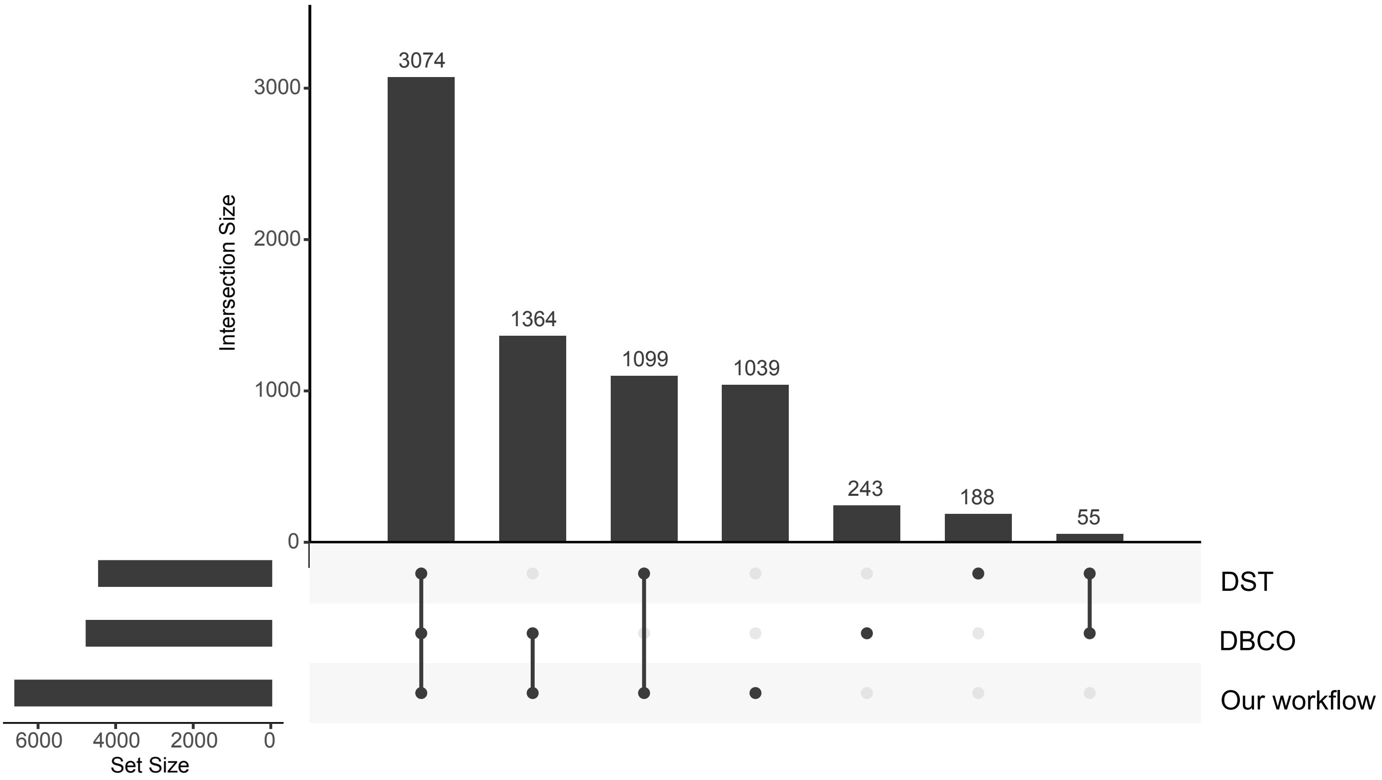

Supplementary Fig. 3: Specifically enriched MetRS* 8661 PDAC cell-derived proteins with different enrichment methods, related to Fig. 1.
Exclusively identified and overlap of specifically enriched protein groups with DST-enrichment, DBCO-enrichment, and our improved enrichment workflow.

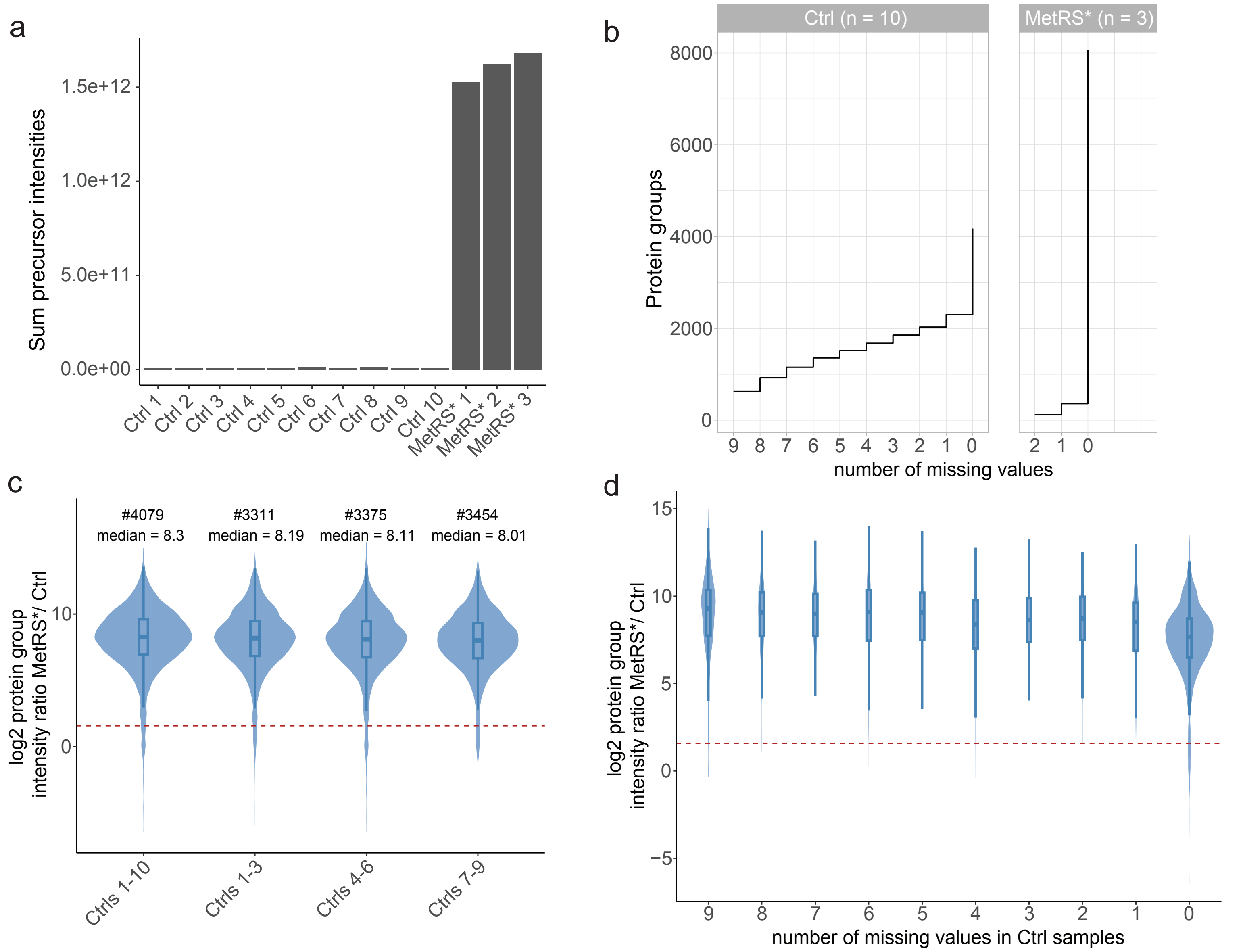

Supplementary Fig. 4: Technical reproducibility of enrichment specificity controls.
MetRS*-expressing (n = 3) or wild-type (Ctrl) (n = 10) 8661 PDAC cells (1 × 10^7 cells per sample) were grown for 8 h in Met-depleted medium supplemented with 4 mM Anl, and processed with our Anl enrichment workflow. **a)** Sum of identified precursor intensities per sample. **b)** Data completeness of identified protein groups in MetRS* and Ctrl samples. **c)** Protein intensity ratios between MetRS* and Ctrl samples analyzed using all measured Ctrl replicates or subsets of 3 Ctrl replicates. **d)** Protein intensity ratios between MetRS* and Ctrl samples seperated by the number of missing values in Ctrl samples.

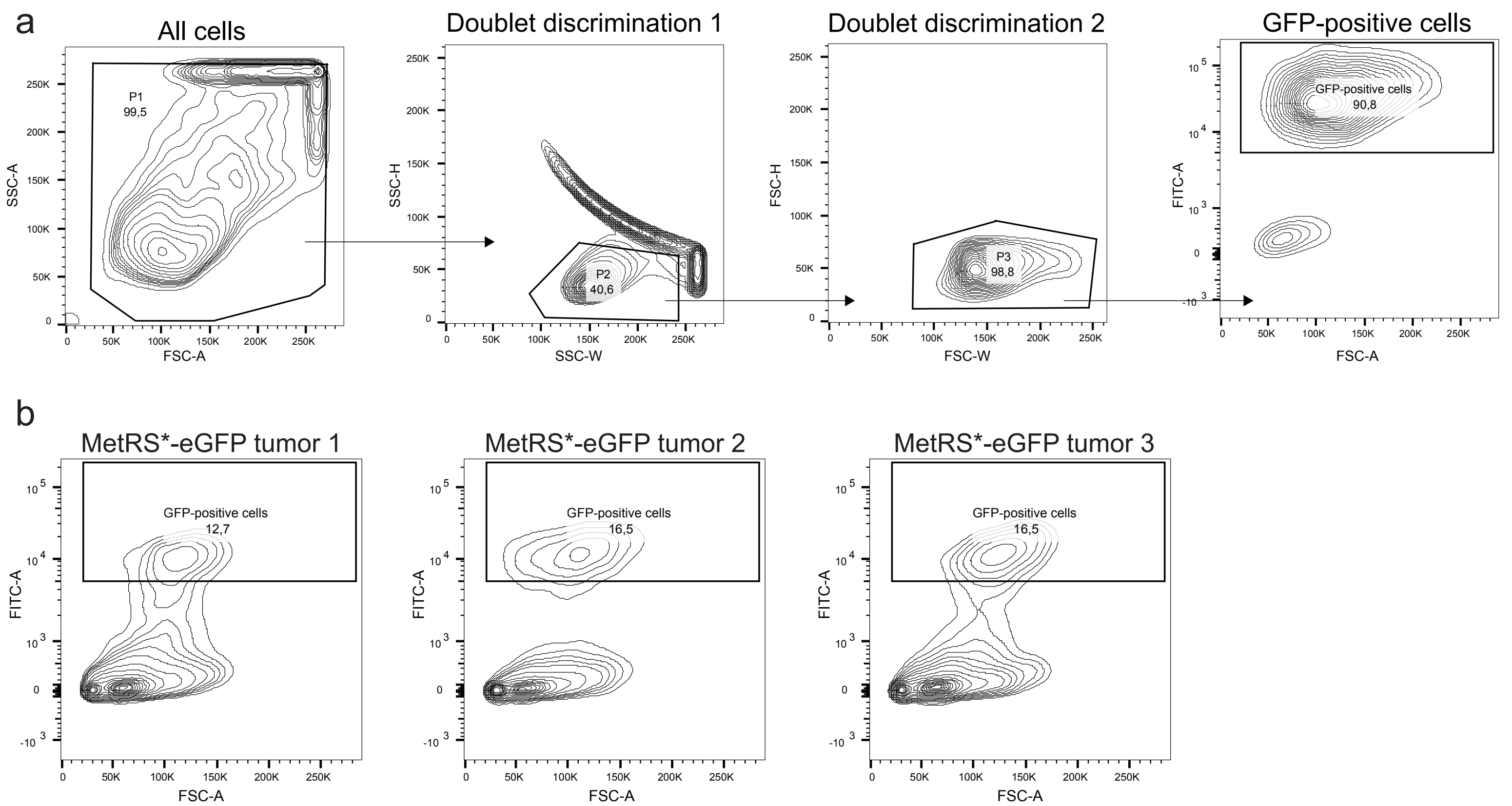

Supplementary Fig 5: Flow cytometry analysis of MetRS*-eGFP 8661 cells, related to Fig. 2.
a) Gating strategy for MetRS* eGFP co-expressing 8661 PDAC cells and evaluation of eGFP-expression before cell transplantation. **b)** Analysis of dissociated tumor cells after orthotopic MetRS*-eGFP 8661 PDAC cell transplantation, tumor growth and Anl labeling (*n* = 3, biological replicates).

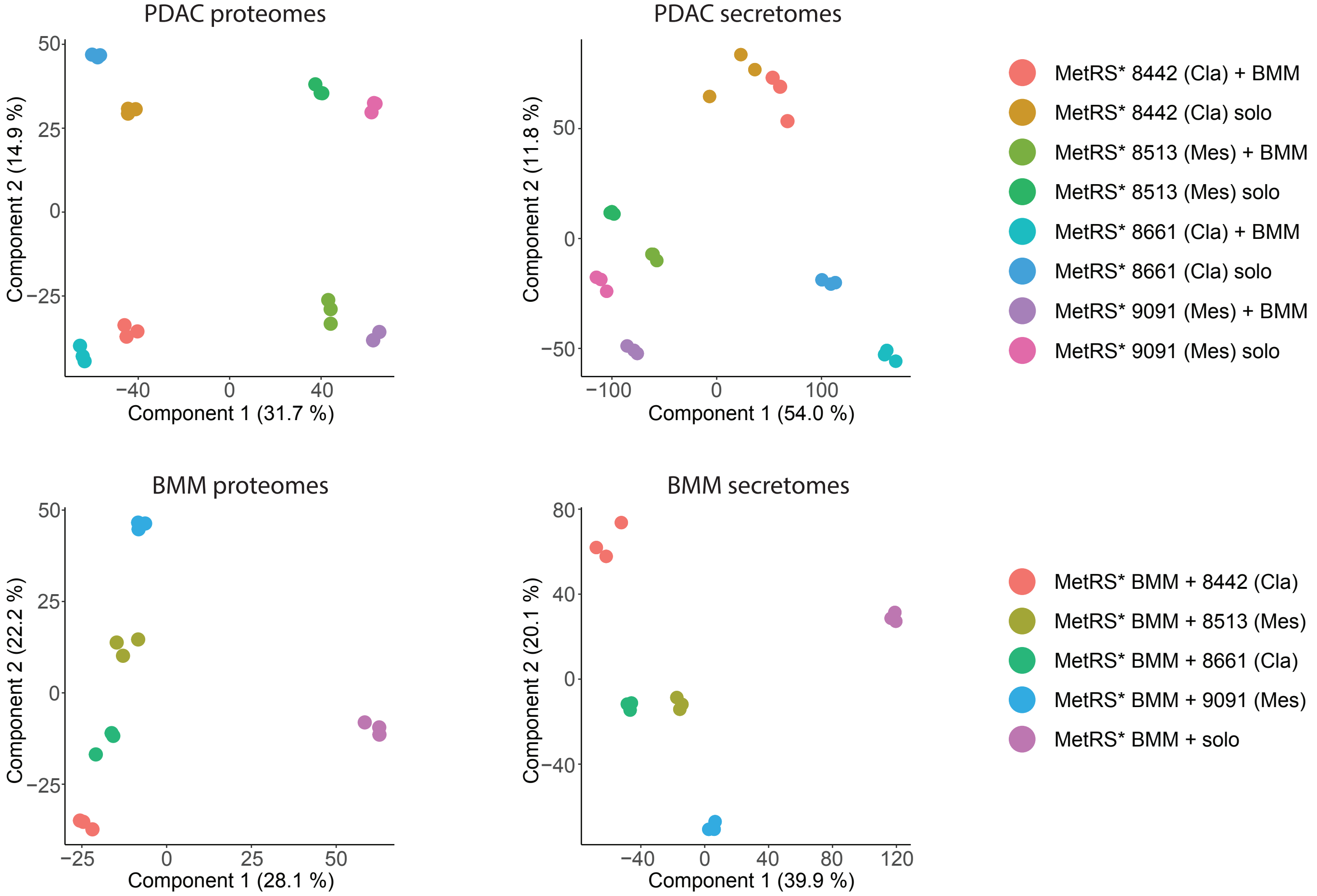

Supplementary Fig 6: Principal component analysis (PCA) of cell-selective proteomics samples from BMMs and PDAC cells in co-culture and isolation *in vitro*, related to Fig. 3.
PCA score plots of the first two principle components of all replicates from global proteome and secretome experiments after filtering for specifically enriched protein groups.

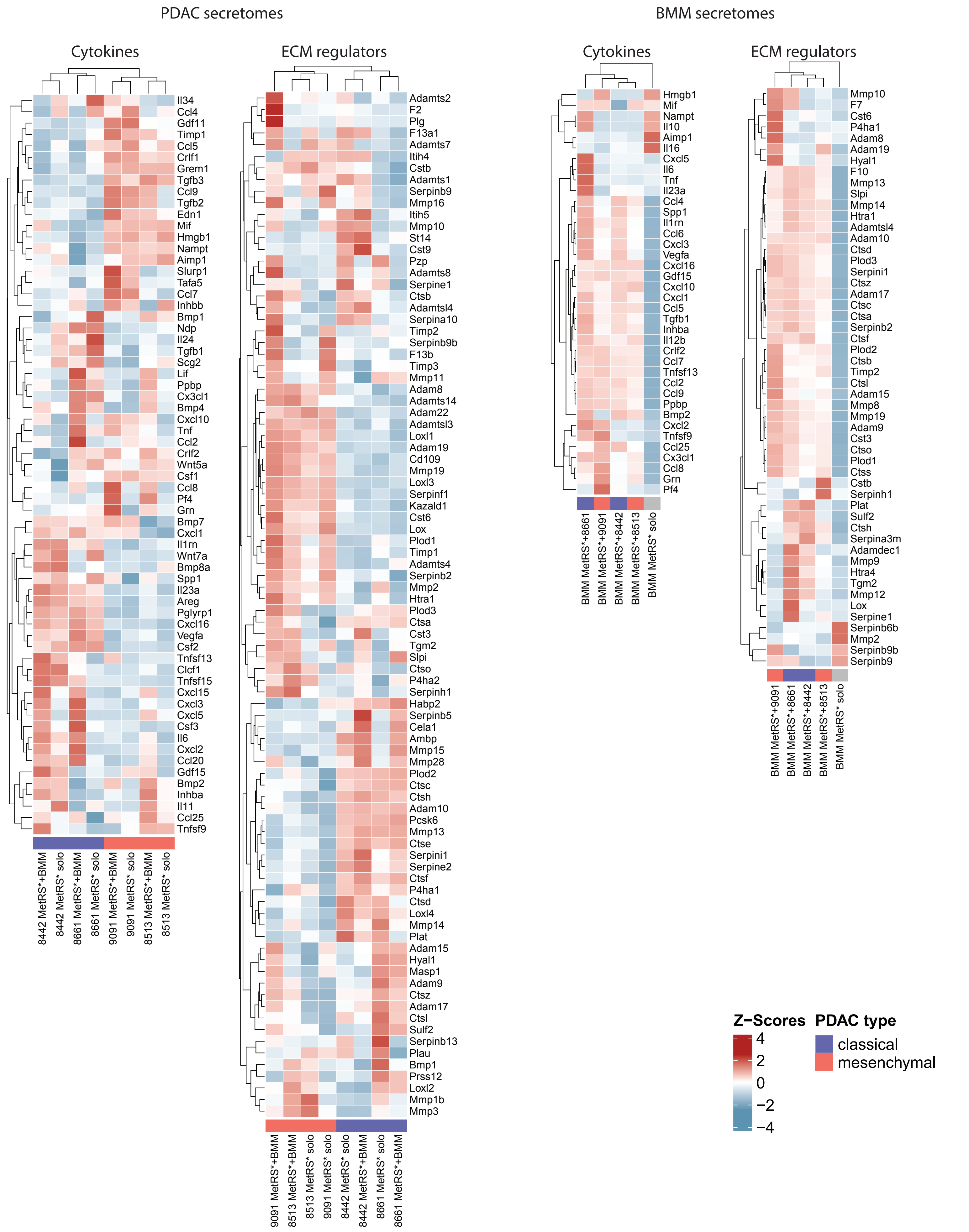

Supplementary Fig 7: Heatmaps of cell-selectively secreted proteins in co-culture experiments, related to Fig. 3. PDAC- and BMM-released proteins with cytokine function or ECM regulator function (according to Naba *et al.*[75]) and significant abundance differences between PDAC subtypes and culture conditions (ANOVA, FDR = 0.05, S0 = 0.1).

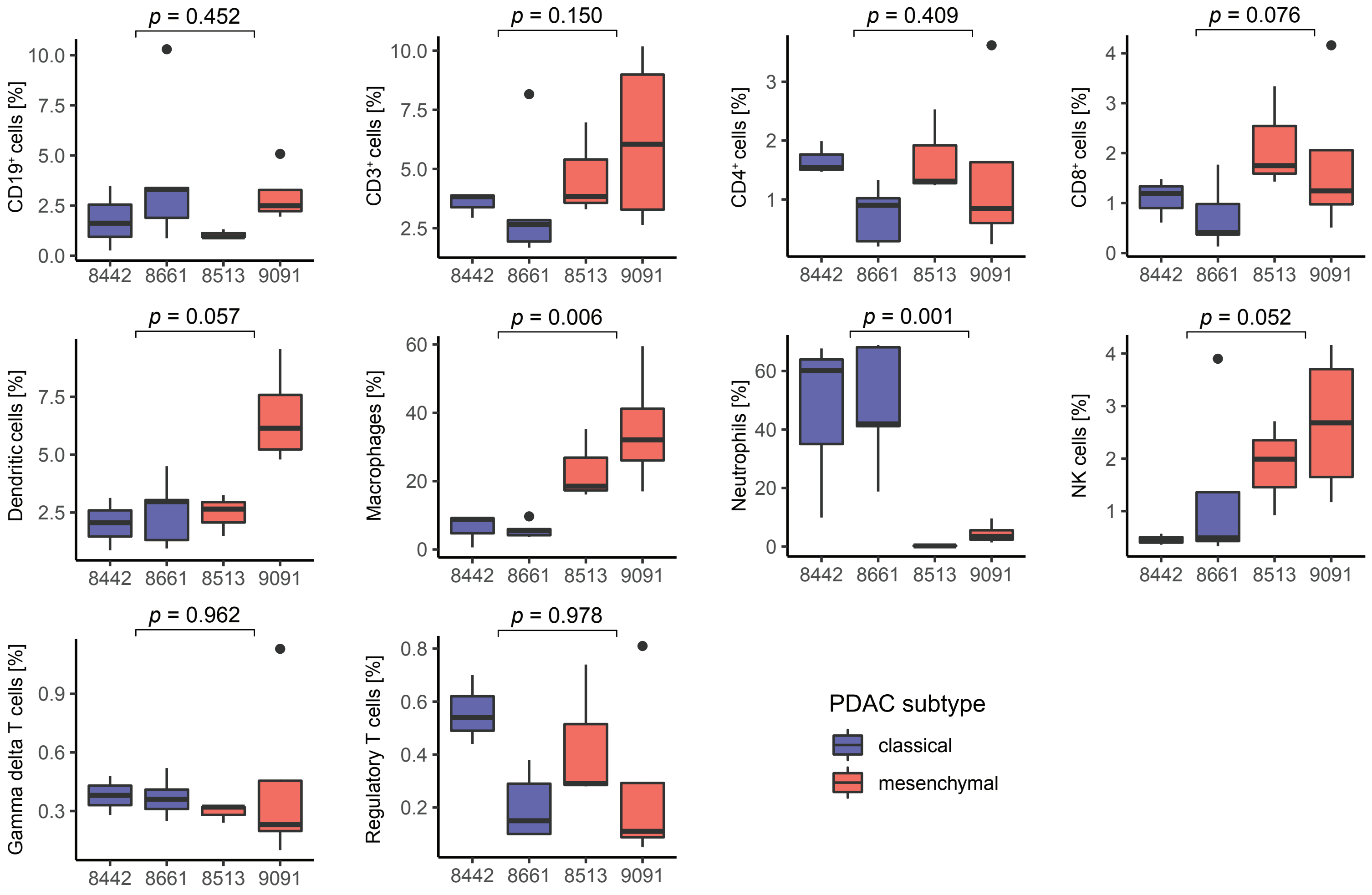

Supplementary Fig. 8: Immunophenotyping of PDAC tumors by flow cytometry, related to Fig. 4.
Cell numbers indicated as % of CD45+ cells. P-values were determined by a two-sided Welch's t-test (8442: *n* = 3, 8661: *n* = 5, 8513: *n* = 3, 9091: *n* = 4; biological replicates) between classical and mesenchymal tumors.

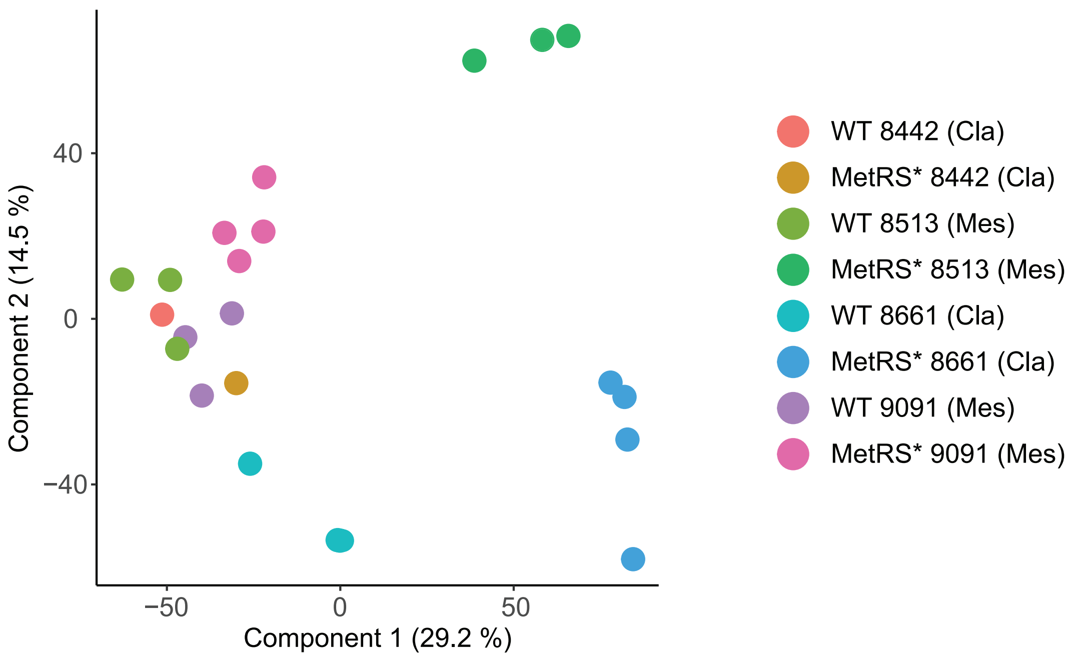

Supplementary Fig. 9: PCA of PDAC tumor-bearing mouse serum samples, related to Fig. 6.
PCA score plots of the first two principle components of all replicates including WT (Ctrl) PDAC samples before filtering MetRS*-samples for specifically enriched protein groups.
