# Supplementary material for: Cell-selective proteomics segregates pancreatic cancer subtypes by extracellular proteins in tumors and circulation
Source: Nat Commun. 2023 May 8;14:2642. doi: 10.1038/s41467-023-38171-8 (PMC10167354; doi:10.1038/s41467-023-38171-8)
Supplement: Supplementary file 3 — Reporting Summary [file 41467_2023_38171_MOESM3_ESM.pdf]

nature portfolio

Corresponding author(s): Felix Meissner

Last updated by author(s): Mar 26, 2023

Reporting Summary

Nature Portfolio wishes to improve the reproducibility of the work that we publish. This form provides structure for consistency and transparency in reporting. For further information on Nature Portfolio policies, see our Editorial Policies and the Editorial Policy Checklist.

Statistics

For all statistical analyses, confirm that the following items are present in the figure legend, table legend, main text, or Methods section.

n/a	Confirmed	
☐	☒	The exact sample size (*n*) for each experimental group/condition, given as a discrete number and unit of measurement
☐	☒	A statement on whether measurements were taken from distinct samples or whether the same sample was measured repeatedly
☐	☒	The statistical test(s) used AND whether they are one- or two-sided<br>*Only common tests should be described solely by name; describe more complex techniques in the Methods section.*
☒	☐	A description of all covariates tested
☐	☒	A description of any assumptions or corrections, such as tests of normality and adjustment for multiple comparisons
☐	☒	A full description of the statistical parameters including central tendency (e.g. means) or other basic estimates (e.g. regression coefficient) AND variation (e.g. standard deviation) or associated estimates of uncertainty (e.g. confidence intervals)
☐	☒	For null hypothesis testing, the test statistic (e.g. *F*, *t*, *r*) with confidence intervals, effect sizes, degrees of freedom and *P* value noted<br>*Give P values as exact values whenever suitable.*
☒	☐	For Bayesian analysis, information on the choice of priors and Markov chain Monte Carlo settings
☒	☐	For hierarchical and complex designs, identification of the appropriate level for tests and full reporting of outcomes
☒	☐	Estimates of effect sizes (e.g. Cohen's *d*, Pearson's *r*), indicating how they were calculated

Our web collection on statistics for biologists contains articles on many of the points above.

Software and code

Policy information about availability of computer code

Data collection	Xcalibur, Thermo Scientific (4.4.16.14)
Data analysis	MaxQuant (version 2.0.1.0.); DIA-NN (version 1.8); Perseus (version 1.6.10.43); R (version 4.1.2); FlowJo (version 10.6.2)

For manuscripts utilizing custom algorithms or software that are central to the research but not yet described in published literature, software must be made available to editors and reviewers. We strongly encourage code deposition in a community repository (e.g. GitHub). See the Nature Portfolio guidelines for submitting code & software for further information.

Data

Policy information about availability of data

All manuscripts must include a data availability statement. This statement should provide the following information, where applicable:

- Accession codes, unique identifiers, or web links for publicly available datasets
- A description of any restrictions on data availability
- For clinical datasets or third party data, please ensure that the statement adheres to our policy

The mass spectrometry proteomics data have been deposited to the ProteomeXchange Consortium (http://proteomecentral.proteomexchange.org) via the PRIDE partner repository with the dataset identifier PXD040084. Source data are provided with this paper. MS spectra were matched against the human (June 2022, 79,276 entries) or mouse (January 2022, 55,105 entries) UniProt FASTA database.

Human research participants

Policy information about studies involving human research participants and Sex and Gender in Research.

Reporting on sex and gender	N/A
Population characteristics	N/A
Recruitment	N/A
Ethics oversight	N/A

Note that full information on the approval of the study protocol must also be provided in the manuscript.

Field-specific reporting

Please select the one below that is the best fit for your research. If you are not sure, read the appropriate sections before making your selection.

☒ Life sciences ☐ Behavioural & social sciences ☐ Ecological, evolutionary & environmental sciences

For a reference copy of the document with all sections, see nature.com/documents/nr-reporting-summary-flat.pdf

Life sciences study design

All studies must disclose on these points even when the disclosure is negative.

Sample size	No sample size calculation was performed. The sample sizes were chosen based on our previous published and unpublished data that have been statistically evaluated, and preliminary experiments that defined the adequate number of samples to consistently identify differences between groups.<br><br>All technical benchmarking experiments (except offline fractionation) and in vitro PDAC experiments were done in workflow triplicates. Offline fractionation of samples for acquisition method comparison was performed without replicates, because the purpose was to generate an identification benchmark and no further quantitative or statistical analysis of these samples. In vivo experiments were done with a minimum of three biologically independent replicates per PDAC subtype (mesenchymal or classical).
Data exclusions	No data was excluded.
Replication	For all experiments with statistical analyses, at least 3 replicates were used, exact n numbers are indicated in figure legends. All attempts at replication were successful.
Randomization	For the experiments described in this manuscript randomization was not possible/appropriate. There were no physical attributes that influenced whether animals were used for control or test groups. All models (tumors or cells in vitro) have been characterized, we did not randomly pick selected ones for further analysis. For orthotopic implantation we sex-matched cell lines and mice.
Blinding	The processing of the samples and their analysis (Anl-protein enrichment, proteomics sample preparation and flow cytometry) were performed in a blinded fashion.

Reporting for specific materials, systems and methods

We require information from authors about some types of materials, experimental systems and methods used in many studies. Here, indicate whether each material, system or method listed is relevant to your study. If you are not sure if a list item applies to your research, read the appropriate section before selecting a response.

Materials & experimental systems

n/a	Involved in the study	
☐	☒	Antibodies
☐	☒	Eukaryotic cell lines
☒	☐	Palaeontology and archaeology
☐	☒	Animals and other organisms
☒	☐	Clinical data
☒	☐	Dual use research of concern

Methods

n/a	Involved in the study	
☒	☐	ChIP-seq
☐	☒	Flow cytometry
☒	☐	MRI-based neuroimaging

Antibodies

Antibodies used	For flow cytometry:<br>CD4 BUV805 (Clone GK1.5, 1:100, BD Biosciences, Cat #564922); CD3εBUV395 (Clone 145-2C11, 1:20, BD Biosciences, Cat #563565); CD11c BUV737 (Clone HL3, 1:30, BD Biosciences, Cat #564986); NK1.1 BUV395 (Clone PK136, 1:25, BD Biosciences, Cat #564144); CD8a BV785 (Clone 53-6.7, 1:100, Biolegend, Cat #100749); CD45 PerCP Cy5.5 (Clone I3/2.3, 1:100, Biolegend, Cat #147705); CD19 FITC (Clone 6D5, 1:100, Biolegend, Cat #115505); EpCAM APC/AF647 (Clone G8.8, 1:200, Biolegend, Cat #118212); Ly6C BV785 (Clone HK1.4, 1:200, Biolegend, Cat #128041); CD11b BV650 (Clone M1/70, 1:100, Biolegend, Cat #101239); F4/80 BV421/PB (Clone BM8, 1:30, Biolegend, Cat #123131); Ly6G PE (Clone 1A8, 1:200, Biolegend, Cat #127607); CD68 APC-CY7 (Clone FA-11, 1:20, Biolegend, Cat #137023); TruStain FcX CD16/32 (Clone 93, 1:100, Biolegend, Cat #101320); CD62L PE (Clone MEL-14, 1:500, Biolegend, Cat #104407)
Validation	All antibodies used in this study were validated for the use in mouse samples by the manufacturers and adequate controls were included (positive and negative controls).<br><br>Detailed information can be found on manufacturers' websites:<br>For flow cytometry:<br>CD4 BUV805: https://www.bdbiosciences.com/en-us/products/reagents/flow-cytometry-reagents/research-reagents/single-color-antibodies-ruo/buv805-rat-anti-mouse-cd4.612900 (replacement for #564922; additional info can be found here: https://www.citeab.com/antibodies/2409851-564922-buv805-rat-anti-mouse-cd4)<br>CD3e BUV395: https://www.bdbiosciences.com/en-us/products/reagents/flow-cytometry-reagents/research-reagents/single-color-antibodies-ruo/buv395-hamster-anti-mouse-cd3e.563565<br>CD11c BUV737: https://www.bdbiosciences.com/en-us/products/reagents/flow-cytometry-reagents/research-reagents/single-color-antibodies-ruo/buv737-hamster-anti-mouse-cd11c.612796 (replacement for #564986; additional info can be found here: https://www.citeab.com/antibodies/3288721-564986-buv737-hamster-anti-mouse-cd11c?des=2b0639313c9c35c8)<br>NK1.1 BUV395: https://www.bdbiosciences.com/en-us/products/reagents/flow-cytometry-reagents/research-reagents/single-color-antibodies-ruo/buv395-mouse-anti-mouse-nk-1-1.564144<br>CD8a BV785: https://www.biolegend.com/en-us/products/brilliant-violet-785-anti-mouse-cd8a-antibody-7957<br>CD45 PerCP Cy5.5: https://www.biolegend.com/en-us/products/percp-cyanine5-5-anti-mouse-cd45-antibody-9793<br>CD19 FITC: https://www.biolegend.com/en-us/products/fitc-anti-mouse-cd19-antibody-1528<br>EpCAM APC/AF647: https://www.biolegend.com/en-us/products/alexa-fluor-647-anti-mouse-cd326-ep-cam-antibody-4973<br>Ly6C BV785: https://www.biolegend.com/en-us/products/brilliant-violet-785-anti-mouse-ly-6c-antibody-11982<br>CD11b BV650: https://www.biolegend.com/en-us/products/brilliant-violet-650-anti-mouse-human-cd11b-antibody-7638<br>F4/80 BV421: https://www.biolegend.com/en-us/products/brilliant-violet-421-anti-mouse-f4-80-antibody-7199<br>Ly6G PE: https://www.biolegend.com/en-us/products/pe-anti-mouse-ly-6g-antibody-4777<br>CD68 APC-CY7: https://www.biolegend.com/en-us/products/apc-cyanine7-anti-mouse-cd68-antibody-13175<br>TruStain FcX CD16/32: https://www.biolegend.com/en-us/products/trustain-fcx-anti-mouse-cd16-32-antibody-5683<br>CD62L PE: https://www.biolegend.com/en-us/products/pe-anti-mouse-cd62l-antibody-386?GroupID=BLG10670

Eukaryotic cell lines

Policy information about cell lines and Sex and Gender in Research

Cell line source(s)	293T cells were obtained from ATCC (CRL-3216). The primary mouse cell lines were isolated from genetically engineered mouse models as previously described in Mueller et al., 2018. Primary mouse PDAC cell lines that were used: 8661, 8442, 9091, 8513
Authentication	The murine cell lines were authenticated through genotyping PCR.
Mycoplasma contamination	All cell lines were routinely checked for mycoplasma contamination and tested negative.
Commonly misidentified lines<br>(See ICLAC register)	No misidentified cell lines according to the ICLAC register were used in this study.

Animals and other research organisms

Policy information about studies involving animals; ARRIVE guidelines recommended for reporting animal research, and Sex and Gender in Research

Laboratory animals	For orthotopic transplantations experiments of PDAC cells, primary murine pancreatic cancer cell lines derived from C57BL/6 mice, the employed animals were on a C57BL/6 background. All animals presented between 8 and 12 weeks of age when the experiments were performed. LysM-Cre/MetRS* mice on a C57Bl6/J background were used to isolate bone marrow derived macrophages. We used LysM-Cre/MetRS* mice between 6-12 months of age.<br>All animals were kept in a dedicated facility, with a light-dark cycle or 12:12 hours, a housing temperature between 20 and 24°C and a relative air humidity of 55%.
Wild animals	No wild animals were used in the study.
Reporting on sex	PDAC cell lines isolated from female endogenous mice were transplanted for the study in female recipients, vice versa for male mice.

Field-collected samples

No field-collected samples were used in the study.

Ethics oversight

The animal studies comported the use of mice and were performed in compliance with the ARRIVE guidelines, moreover all animal studies were conducted on compliance with European guidelines for the care and use of laboratory animals and were approved by the Institutional Animal Care and Use Committees (IACUC) of the local authorities of Technische Universität München and the Regierung von Oberbayern.

Note that full information on the approval of the study protocol must also be provided in the manuscript.

Flow Cytometry

Plots

Confirm that:

- [x] The axis labels state the marker and fluorochrome used (e.g. CD4-FITC).
- [x] The axis scales are clearly visible. Include numbers along axes only for bottom left plot of group (a 'group' is an analysis of identical markers).
- [x] All plots are contour plots with outliers or pseudocolor plots.
- [x] A numerical value for number of cells or percentage (with statistics) is provided.

Methodology

Sample preparation

Fresh tumor samples were minced using a scalpel and enzymatically digested with the tumor dissociation kit (Miltenyi #130-096-730) in DMEM medium (Sigma, #D5796-500 mL) for 40 min at 37°C with agitation. The cell suspension was strained through a 100 µm strainer, spun down and resuspended in 2% FCS/PBS. Cells were blocked for 10 min on ice with anti-mouse CD16/CD32 FC block (Biolegend, 1:100) and stained with Zombie Aqua Fixable Viability Kit (Biolegend, 1:500) (for 10 min on ice) and the antibody cocktails for acquisition of adaptive immune cells as well as innate immune cells (for 30 min on ice).

For sorting of EGFP-positive PDAC cells:
PDAC cell lines we deattached with trypsin, washed three times with ice-cold PBS and filtered through 30 µm mesh before acquisition. Fresh tumor samples were dissociated as described above. Debris removal solution (Miltenyi #130-109-398) was used to discard cell debris from the cell suspension and removal of dead cells was performed using the dead cell removal kit (Miltenyi #130-090-101). The enriched fraction of live cells was collected in ice-cold 2% FCS/PBS buffer and filtered through a 30 µm mesh before acquisition

Instrument

BD LSRFortessa (Immunophenotyping); BD FACS Aria Fusion (Sorting of GFP-positive PDAC cells)

Software

FlowJo software (Version 10.6.2)

Cell population abundance

EGFP-positive PDAC cells in vitro: 80-90% of total cells
EGFP-positive PDAC cells in vivo: < 20% of total cells

Gating strategy

FSC and SSC gates were used to identify cells and exclude doublets. Live/dead cells were discriminated by Zombie Aqua staining (staining of dead cells). Live cells were further analyzed for cell types of interes, which can be analyzed by the following marker:

Neutrophils: CD45+ LygG+ CD11b+
Macrophages: CD45+ LygG- CD11b+ F4/80+
Dendritic cells: CD45+ LygG- F4/80- CD11c+
NK cells: CD45+ LygG- F4/80- CD11c- NK1.1+
T cells: CD45+ CD3+
B cells: CD45+ CD19+
CD4+ T cells: CD45+ CD3+ CD4+
CD8+ T cells: CD45+ CD3+ CD8a+

For sorting of EGFP-positive PDAC cell fraction FSC and SSC gates were used to identify cells and exclude doublets. EGFP-positive cells were sorted.

- [x] Tick this box to confirm that a figure exemplifying the gating strategy is provided in the Supplementary Information.
