# Supplementary material for: Cell-selective proteomics segregates pancreatic cancer subtypes by extracellular proteins in tumors and circulation
Source: Nat Commun. 2023 May 8;14:2642. doi: 10.1038/s41467-023-38171-8 (PMC10167354; doi:10.1038/s41467-023-38171-8)
Supplement: Supplementary file 4 — Description of Additional Supplementary Files [file 41467_2023_38171_MOESM4_ESM.docx]

**Description of Additional Supplementary Files**

File Name: Supplementary Data 1
Description: Cell-selective quantitative proteomics and secretomics analyses of PDAC cells and macrophages in co-culture and isolation (related to Fig. 3 and 4).

File Name: Supplementary Data 2
Description: Cancer cell-selective quantitative proteomics analyses of mouse tumors formed after orthotopic transplantation of PDAC cells (related to Fig. 5).

File Name: Supplementary Data 3
Description: Quantitative analyses of cancer cell-derived proteins in the serum of PDAC tumor-bearing mice (related to Fig. 6).

File Name: Supplementary Data 4
Description: Output of annotation enrichment tests performed in this study (related to Fig. 2, 3 and 5).
